# DeepRaman: Implementing surface-enhanced Raman scattering together with cutting-edge machine learning for the differentiation and classification of bacterial endotoxins

**DOI:** 10.1016/j.heliyon.2025.e42550

**Published:** 2025-02-08

**Authors:** Samir Brahim Belhaouari, Abdelhamid Talbi, Mahmoud Elgamal, Khadija Ahmed Elmagarmid, Shaimaa Ghannoum, Yanjun Yang, Yiping Zhao, Susu M. Zughaier, Halima Bensmail

**Affiliations:** a Hamad Bin Khalifa University, Department of Computer Sciences and Engineering, Doha, Qatar; b Department of Basic Medical Sciences, College of Medicine, Qatar University, Doha, Qatar; c Weill Cornell Medicine-Qatar, Education City, Qatar Foundation, Doha, Qatar; d Qatar Computing Research Institute, Qatar Center for Artificial Intelligence, Hamad Bin Khalifa University, Qatar; e University of Georgia, College of Engineering, Athens, GA, USA; f University of Georgia, Department of Physics and Astronomy, Athens, GA, USA

**Keywords:** Raman spectroscopy, CNN, Fourier transform, Progressive fourier transform, Scalogram, SVM, k-NN, ImageNet, Dense CNN

## Abstract

To classify raw SERS Raman spectra from biological materials, we propose *DeepRaman*, a new architecture inspired by the Progressive Fourier Transform and integrated with the scalogram transformation approach. Unlike standard machine learning approaches such as PCA, LDA, SVM, RF, GBM etc, *DeepRaman* functions independently, requiring no human interaction, and can be used to much smaller datasets than traditional CNNs. Performance of DeepRaman on 14 endotoxins bacteria and on a public data achieved an extraordinary accuracy of 99 percent. This provides exact endotoxin classification and has tremendous potential for accelerated medical diagnostics and treatment decision-making in cases of pathogenic infections.

**Background:**

Bacterial endotoxin, a lipopolysaccharide exuded by bacteria during their growth and infection process, serves as a valuable biomarker for bacterial identification. It is a vital component of the outer membrane layer in Gram-negative bacteria. By employing silver nanorod-based array substrates, surface-enhanced Raman scattering (SERS) spectra were obtained for two separate datasets: Eleven endotoxins produced by bacteria, each having an 8.75 pg average detection quantity per measurement, and three controls chitin, lipoteichoic acid (LTA), bacterial peptidoglycan (PGN), because their structures differ greatly from those of LPS.

**Objective:**

This study utilized various classical machine learning techniques, such as support vector machines, k-nearest neighbors, and random forests, in conjunction with a modified deep learning approach called DeepRaman. These algorithms were employed to distinguish and categorize bacterial endotoxins, following appropriate spectral pre-processing, which involved novel filtering techniques and advanced feature extraction methods.

**Result:**

Most traditional machine learning algorithms achieved distinction accuracies of over 99 percent, whereas *DeepRaman* demonstrated an exceptional accuracy of 100 percent. This method offers precise endotoxin classification and holds significant potential for expedited medical diagnoses and therapeutic decision-making in cases of pathogenic infections.

**Conclusion:**

We present the effectiveness of *DeepRaman*, an innovative architecture inspired by the Progressive Fourier Transform and integrated with the scalogram transformation method, in classifying raw SERS Raman spectral data from biological specimens with unparalleled accuracy relative to conventional machine learning algorithms. Notably, this Convolutional Neural Network (CNN) operates autonomously, requiring no human intervention, and can be applied with substantially smaller datasets than traditional CNNs. Furthermore, it exhibits remarkable proficiency in managing challenging baseline scenarios that often lead to failures in other techniques, thereby promoting the broader clinical adoption of Raman spectroscopy.

## Abbreviations:

AgNR: Angle of Silver Nanorods
ANN: Artificial Neural Networks
AirPLS: Adaptive Iteratively Reweighted Penalized Least Squares
AsLS: Asymmetric Least Square smoothing
CNN: Convolutional Neural Network
CFT: Continuous Fourier Transform
DenseCNN: Densely Connected 3D Convolution Neural Network
DenseNet: Dense Convolutional Network
DFT: Discrete Fourier Transform
FFT: Fast Fourier Transform
HCA: Hierarchical Cluster Analysis
k-NN: k-Nearest Neighbor
LDA: Linear Discriminant Analysis
LPS: lipopolysaccharides
LTA: Lipoteichoic acid
OAD: Oblique Angle Deposition
PCA: Principal Component Analysis
PFT: Progressive Fourier Transform
PGN: Peptidoglycan
PLS-DA: Partial Least Square Discriminant Analysis
ResNet: Residual Network
RF: Random Forest
SERS: Surface-Enhanced Raman Spectroscopy
SNR: Signal-to-Noise Ratio
SVM: Support Vector Machine.
TLR4: Toll-like receptor 4

## 1 Introduction

Bacterial infections pose significant health risks, emphasizing the need for early detection and intervention. While bacterial culture remains the gold standard for diagnosis, indirect methods target indicators like metabolites, pigments, endotoxins, and small molecules such as pyoverdine and pyocyanin [1,2]. Among these, lipopolysaccharides (LPS), key endotoxins in Gram-negative bacteria, play a critical role by triggering TLR4-mediated immune responses and contributing to sepsis and cytokine storms [3]. Even trace amounts of circulating LPS are linked to septic shock and mortality [4,5]. Rapid endotoxin detection is thus crucial for clinical diagnosis and treatment. Recent advancements in nanotechnology have enabled biosensors using electrical, chemiluminescent, and fluorescence techniques to detect trace endotoxins [6,7]. Surface-Enhanced Raman Spectroscopy (SERS) has emerged as a powerful tool for multiplexed detection, offering molecular-level sensitivity and broad applications, including diagnostics and endotoxin fingerprinting [[8], [9], [10], [11]].

Nonetheless, a major challenge in SERS endotoxin detection is the identification of distinct SERS spectral features. Many endotoxins share similar chemical structures, resulting in highly similar SERS spectra. Consequently, a range of statistical methods, encompassing both supervised and unsupervised learning [[12], [13], [14], [15]], is employed to classify these spectra. Moreover, SERS spectra are inherently multivariate data, necessitating the application of chemometric analysis to reduce data dimensionality and optimize variance across spectral fingerprints for microorganism differentiation. Traditional chemometric methods, however, face increasing challenges in differentiating SERS spectra due to several factors. Vibrational spectra from microbial systems or individual cellular often exhibit a Low Signal-to-Noise Ratio (SNR), increasing the complexity of data analysis. Additionally, artifacts introduced during SERS experiments, including fluorescence emissions, thermal noise, optical filter quality, and spectrometer calibration precision, necessitate rigorous preprocessing to eliminate such anomalies before they can be employed for diagnostic purposes. Preprocessing techniques encompass cosmic ray reduction, spectral denoising, and baseline modifications.

Raman spectroscopy analyzes laser light interactions to produce optical fingerprints, while faster observations yield noisier spectra, requiring machine learning for interpretation. SERS, known for its sensitivity and specificity, aids in endotoxin detection but faces challenges in distinguishing overlapping spectra due to similar chemical structures, as noted in both direct and indirect approaches [16,17].

Various methods are used for SERS spectra classification, including Principal Component Analysis (PCA) and Hierarchical Cluster Analysis (HCA) for unsupervised learning, and Support Vector Machines (SVM), Linear Discriminant Analysis (LDA), k-Nearest Neighbors (k-NN), Partial Least Squares Discriminant Analysis (PLS-DA), and Random Forest (RF) for supervised learning. PLS-DA excels in handling high-dimensional data, aiding in the identification of spectral differences. However, the complexity of SERS spectra, stemming from diverse molecular contributions, and the low signal-to-noise ratio (SNR) in vibrational spectra can obscure critical features, complicating classification in complex biological systems [18,19]. For baseline correction, methods such as polynomial fitting and least squares are employed to handle signal distortion and reduce discrepancies. Polynomial methods fit a baseline by iteratively removing signal peaks or applying linear constraints, though they require determining an optimal degree of freedom, which can lead to issues if not set appropriately. Least square-based methods, such as asymmetric least square (AsLS) smoothing and Adaptive Iteratively Reweighted Penalized Least Squares (airPLS) method, aim to minimize discrepancies between expected baselines and real signals, though these algorithms also involve tuning parameters like penalty weights, requiring human input for satisfactory results [[20], [21], [22], [23], [24], [25], [26], [27], [28], [29], [30], [31], [32], [33], [34], [35], [36], [37], [38], [39], [40], [41]].

Recent advancements have utilized Convolutional Neural Networks (CNNs) for direct classification of raw Raman spectra and automated denoising and baseline correction, leveraging their ability to extract global and local spectral features. Liu et al. demonstrated superior classification accuracy of chemical species in minerals using CNNs compared to traditional methods like LDA, SVM, and k-NN [42]. Wahl et al. applied CNNs for automated baseline correction, outperforming conventional techniques [43]. CNNs have also been applied to classify diverse biological samples, including *E. coli* strains and cancer tissues [[44], [45], [46], [47], [48], [49], [50], [51], [52], [53]]. Recent methods include Zhang et al.'s neural network combining variational mode decomposition and bidirectional LSTM [54], Han et al.'s convolutional autoencoder [55], Wang et al.'s back propagation network [56], and Gu et al.'s GAN-enhanced denoising autoencoder [57]. Unfortunately, previous models rely on latent space representations that may not always capture fine-grained spectral details effectively and are designed for unsupervised learning and may not directly optimize for classification accuracy.

Our proposed approach combines CNN and scalogram techniques for Raman spectroscopy classification. CNNs excel in learning hierarchical features, while scalograms provide a compressed, informative representation of spectroscopy data. This combination helps reduce dimensionality, improve generalization, and enhance classification accuracy, especially in noisy environments. Scalograms offer visual insights into predictions, making the approach more interpretable. Compared to ResNet alone, our method captures a broader range of features, making it effective for complex, noisy data.

In this reasearch, we present a method that provides explicit time-frequency representations (via progressive fast transform), preserving essential spectral and temporal features crucial for Raman spectroscopy, and facilitates feature selection and efficient gradient flow, enabling robust classification even with complex, noisy data. Our mode integrates feature extraction, undergo transformation into scalogram images, on which a dense CNN architecture is trained to yield an algorithm referred to as *DeepRaman*.

Our contribution is summarized as the following.

1. We enhance the signal using an advanced technique that calculates the average between two signals, resulting in a signal with reduced high-frequency content compared to the original signals. This approach is particularly effective in managing difficult baseline scenarios that frequently cause failures.
2. Next, we convert the processed signal into scalogram images, providing a detailed time-frequency decomposition representation.
3. These images are then fed into a pre-trained CNN model (DenseCNN) to obtain learned aspects for categorization purposes.

## 2 Method

### 2.1 Data preparation of bacterial endotoxins

Various chemicals and materials, including sulfuric acid, ammonium hydroxide, hydrogen peroxide, ethyl alcohol, silver, titanium pellets, and pure water, were used for glass slide cleaning, evaporation, and experiments. Array of silver nanorods (AgNR) were fabricated using oblique Angle Deposition (OAD) and are recognized as highly effective SERS substrates, involving the deposition of Ti and Ag films on glass slides followed by rotation and deposition of Ag to achieve the desired arrays under high vacuum conditions. Lipopolysaccharides were isolated from bacterial cells through a hot phenol-water extraction procedure, purified, and various LPS samples were obtained from different bacterial species for study as previously described in details.

(https://pubmed.ncbi.nlm.nih.gov/15845500/and https://pubmed.ncbi.nlm.nih.gov/35686584/)

with S. meliloti being non-pathogenic to humans.

Bacterial endotoxin samples (LPSs) were diluted, dispensed onto AgNR substrates, air-dried, and analyzed using a confocal Raman microscope with specific settings. Technically, the purified bacterial endotoxins and control samples were diluted to 100 μg/mL in pure water. To measure SERS spectra, 2 μL was placed on the AgNR substrate and air-dried at 20 °C, forming an estimated spread area of ∼3.5 mm^2^. SERS spectra were acquired with a confocal Raman microscope (Renishaw InVia, 785 nm excitation, 9 mW at sample, 5 × objective, 10 s exposure). The laser spot (∼1875 μm^2^) probed ∼8.75 pg of sample per measurement. To generate data for each bacterial endotoxin, SERS mappings were collected from 4 to 5 substrates points with at least 300 μm spacing between sites and about 100 spectra were measured per mapping (ref. https://pubmed.ncbi.nlm.nih.gov/35686584/) with S. meliloti being non-pathogenic to humans.

Bacterial endotoxin samples were diluted, dispensed onto AgNR substrates, air-dried, and analyzed using a confocal Raman microscope with specific settings.

A total of 5624 SERS spectra were acquired, derived from 11 bacterial endotoxins, and three controls: chitin, lipoteichoic acid (LTA), and peptidoglycan (PGN).

This section will provide the specifics of the proposed DeepRaman algorithm. The process is outlined as follows: We demonstrate the efficacy of this innovative *DeepRaman* architecture, which is inspired by the Progressive Fourier Transform and combines it with the scalogram transformation method to achieve superior accuracy in classifying raw SERS Raman spectra from biological samples compared to traditional machine learning algorithms. The CNN model described in this work operates autonomously, eliminating the need for human intervention and enabling the use of smaller datasets compared to standard CNNs. Furthermore, it excels in handling challenging baseline scenarios that often lead to failures in other techniques, making it particularly valuable for advancing the broader clinical application of Raman spectroscopy.

The algorithm consists of the following steps: Step 1) Preparing raw spectra. Step 2) Denoising data. Step 3) Transforming the processed data into scalogram images using Progressive Fourier Transform (PFT). Step 4) Feeding the images into a previously trained CNN model (DenseCNN) to obtain extracted features. Step 5) Examining the collected features using k-NN classifier.

**Fig. 1:**
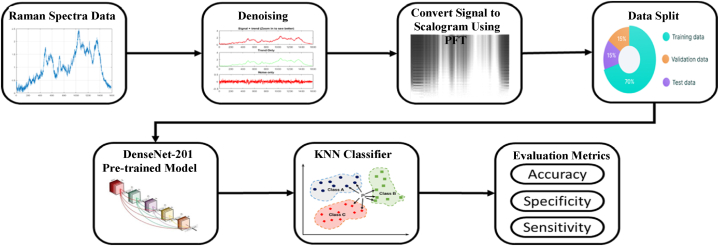
*DeepRaman* process from raw data to classification performance.

A summarized representation of the algorithm can be found in Fig. 1. Next, we will give detailed explanation of the steps.

Step 2 Datasets Preprocessing:

From the signal amplitude variation, upper and lower bound signal will be extracted to form an envelope of the signal respectively. The first average signal between upper and lower signal will have fewer high frequencies compared to the original signal (see Figs. S1–S3 and detailed explanation in supplementary file).

Step 3 Modification of the spectra through Progressive Fourier Transform (PFT)

We propose an innovative time-frequency representation, called Progressive Fourier Transform (PFT), founded on the Fourier Transform. Stated otherwise, we employed this technique to progressively apply the Fourier transform to a sample of signal values, as we zero out the remaining data points in order to transform the values to the frequency domain on the chosen sample until we attain the complete size of the signal values.

**Fig. 2:**
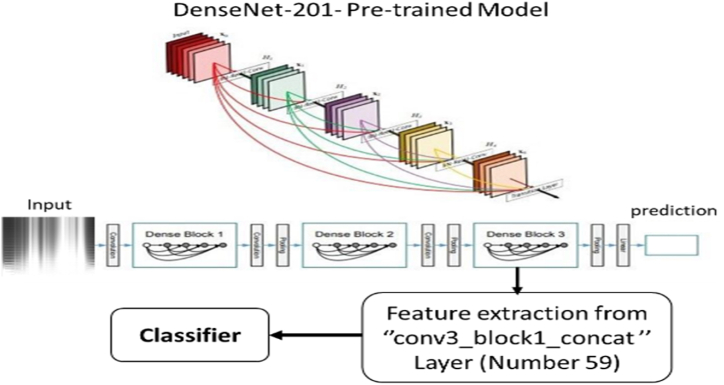
CNN-based architecture applied to transformed scalogram data.

**Fig. 3:**
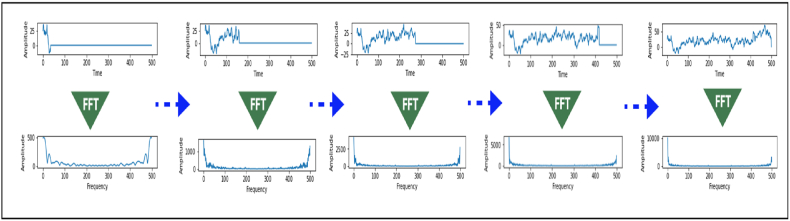
Using the progressive Fourier transform (PFT) to analyze the signal progressively.

Equation (1) defines the equation for the proposed approach. Fig. 2, Fig. 3 illustrate the implementation procedure for this approach.

(1) $$F\left(f,S\right)=\int_{-\infty }^{+\infty }f\left(x\right)1_{x\leq S}e^{-j2\pi \tau }d\tau ,S\in R$$

Each signal will be changed to two-dimension representation, time and frequency representation.

Step 4 Feature Extraction Utilizing Pre-trained Deep Neural Networks and Classification

Within the domain of computer vision applications, the foundation of cutting-edge machine learning models lies in deep learning. The utilization of local convolution filters to extract region-specific information necessitates the incorporation of convolutional neural networks (CNNs), which represent a crucial component within deep neural networks. CNNs hold a unique position in the domain of medical image processing and significantly contribute to the advancement of biomedical research [58,59].

**Fig. 4:**
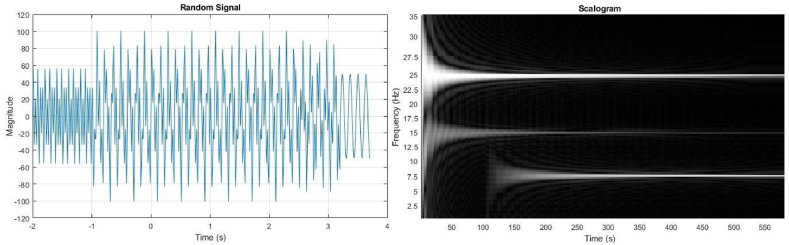
Transforming Signal data to Scalogram.

Within our investigation, we opted for the CNN architecture that boasts superior accuracy, namely DenseNet-201 (Dense Convolutional Network), for feature extraction (Fig. 4). This choice aligns with previous studies that employed scalogram images [60]. In DenseNet, each layer incorporates extra inputs from the previous layers and transmits its own feature maps to all subsequent layers through the process of concatenation.

This design ensures that each layer gains a collective knowledge from its predecessors. Consequently, the network can maintain a leaner and more compact structure, with fewer channels, thanks to the introduction of the growth rate parameter (k), signifying the additional number of channels for each layer. This architectural choice enhances computational and memory efficiency.

Each composition layer in DenseNet follows a pre-activation sequence of Batch Norm (BN) and Rectified Linear Unit ReLU (followed by a 3x3 Convolution operation that yields output feature maps with k channels).

A notable advantage of DenseNet is its ability to generate diverse features, in contrast to ResNet, which tends to produce correlated features. Additionally, DenseNet boasts a considerably smaller size when compared to ResNet [58], a competing architecture.

The algorithm's effectiveness is assessed through a standard training-testing split procedure, a common practice in machine learning. Our dataset is partitioned into two subsets, with 70 % assigned to training and 30 % set aside to testing. This division takes place following the transformation of the Raman signal into scalogram images through the Progressive Fourier Transform (PFT) method. Following this, features derived from the pre-trained CNN's layer activations are obtained and adapted to the k-Nearest Neighbors (k-NN) classifier.

### 2.2 Performance measure

The performance of each model is subsequently evaluated using assessment metrics outlined in equations (2), (3), (4), (5). Among these metrics, TP (True Positive) serves as a critical indicator of the model's ability to accurately predict individual bacterial endotoxins versus all. TN (True Negative) measures the model's ability to accurately predict instances of specific bacterial endotoxins. FP (False Positive) is essential for assessing instances where the prototype erroneously predicts individual bacterial endotoxins versus all, while FN (False Negative) provides a synopsis of the model's incorrect predictions of individual bacterial endotoxins.

(2) $$\text{Accuracy}=\frac{TP+TN}{TP+FP+TN+FN}$$

(3) $$\text{Sensitivity}=\frac{TP}{TP+FN}$$

(4) $$\text{Precision}=\frac{TP}{TP+FP}$$

(5) $$\text{Specificity}=\frac{TN}{TN+FP}$$

## 3 Results

The general approach for detecting and classifying bacterial endotoxins, is portrayed in Fig. 1 by using SERS (Surface-Enhanced Raman Spectroscopy) analysis. The process begins with the compilation of a detailed database of SERS spectra related to bacterial endotoxins, accomplished by collecting spectra from highly sensitive AgNR (silver nanorod array) substrates. Subsequently, baseline correction is applied based on spectral features to yield highly reproducible spectra. Ultimately, the precise differentiation of bacterial endotoxins based on SERS spectra is accomplished by employing classical machine learning methods such as SVM, RF, k-NN, ANN, LDA and our proposed deep learning model *DeepRaman*.

A significant challenge when applying deep learning to Raman spectrum research is the scarcity of benchmark datasets that are available to the general public [55]. Even though deep learning models have been used in recent research on Raman spectrum analysis, the majority of datasets are still private or proprietary [60,61]. As a result, we started by doing a thorough review of current studies being done in the area of Raman spectrum analysis. Initially, we evaluated the algorithm's performance using a publicly available dataset (Covid-19). Subsequently, we conducted an extensive evaluation of *DeepRaman*'s performance on our SERS dataset.

### 3.1 Performance of DeepRaman on Covid dataset

In this part, we examine the effectiveness of our algorithm on a public dataset generated by Raman spectroscopy approach to demonstrate the model's universality.

**Fig. 5:**
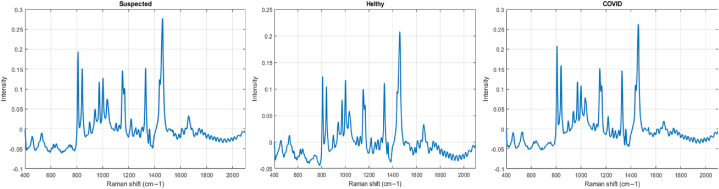
Normalized and baseline corrected spectra. Suspected (left), healthy (middle) and Covid-19 diagnosed (right).

Our study made use of publicly accessible data from a recent publication [62], which served as an preliminary investigation of Raman spectroscopy's main use in COVID-19 screening. The dataset included 177 serum samples that were taken from three distinct groups: 63 patients affected by COVID-19, 59 probable COVID-19 infected patients, and 55 individuals classified as the control group (i.e., healthy individuals). Within the COVID-19 group, there were 58 symptomatic and 5 asymptomatic individuals hired from the Clinical Medical Center for Public Health in Chengdu. The presumed group manifested flu-like symptoms but returned negative results on RT-PCR assays. Serum samples were collected from all individuals by centrifugation at 3000 rpm for 10 min, within 1 h of blood collection, and were subsequently stored at 4C^0^. Raman excitation was performed using a single-mode diode laser with a 785 nm wavelength and a 100-mW power output. The laser intensity for each sample was approximately 70 mW, and Raman spectra were captured within the 600-1800 cm^−1^ range (as illustrated in Fig. 5).

In contrast to Refs. [[58], [59], [60], [61]], our approach encompassed the entire spectrum rather than specific data points that exhibited significant differences in ANOVA.

**Fig. 6:**
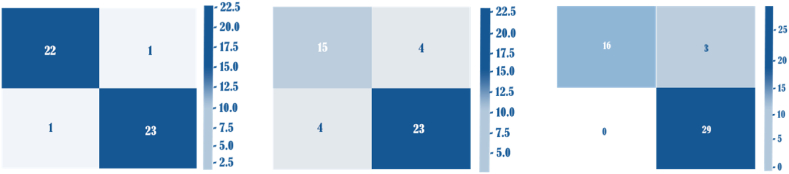
Performance of one versus all using on Covid data. Left figure sumarizes the performace of Covid versus Healthy, middle figure is Suspected versus Covid and right figure is Suspected versus Healthy.

**Fig. 7:**
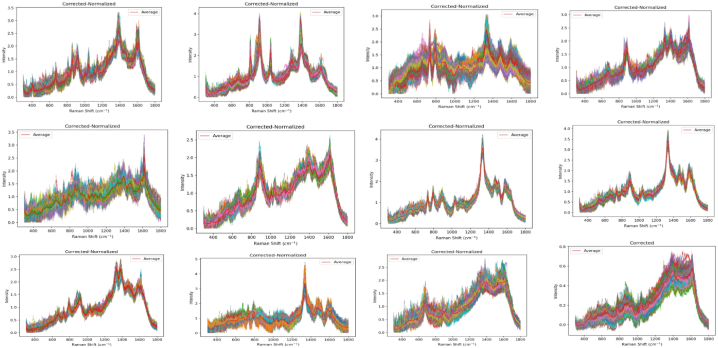
Eleven SERS LPSs and one reference samples and their distribution.

We followed the same evaluation methodology as described in Ref. [62]. Fig. 6 and Table 1 present the mean performance outcomes for a variety of activities across 50 random trials, including numbers for specificity, sensitivity, and accuracy values. Among these tasks, the most challenging one was identifying suspects and healthy participants, as evidenced by the relatively lower performance of SVM (all metrics <82 %). Conversely, *DeepRaman* demonstrated comparatively improved performance in this activity. Remarkably, *DeepRaman* enhanced specificity and accuracy by 10 % and 14 %, correspondingly, when compared to SVM and RamanNet [63] (see Fig. 7).

**Table 1:** Performance of *DeepRaman* for Covid data.

Comparison	Accuracy	Sensitivity	Specificity
**Covid versus healthy**			
*DeepRaman*	0.966 ± 0.001	0.985 ± 0.004	0.923 ± 0.01
SVM	0.910 ± 0.040	0.890 ± 0.070	0.930 ± 0.06
RamanNet	0950 ± 0.000	0.950 ± 0.040	0.960 ± 0.03
**Covid versus suspected**			
*DeepRaman*	0.945 ± 0.002	0.979 ± 0.001	0.859 ± 0.03
SVM	0.870 ± 0.050	0.890 ± 0.080	0.860 ± 0.09
RamanNet	0.930 ± 0.030	0.970 ± 0.040	0.900 ± 0.06
**Healthy versus suspected**			
*DeepRaman*	0.929 ± 0.005	0.736 ± 0.001	0.937 ± 0.02
SVM	0.690 ± 0.050	0.700 ± 0.090	0.660 ± 0.09
RamanNet	0.820 ± 0.060	0.770 ± 0.150	0.870 ± 0.11

### 3.2 Bacteria dataset

The lipopolysaccharides employed in the preparation and purification of bacterial endotoxins were procured by performing a hot phenol-water extraction procedure on bacterial cells [64]. Subsequently, the water phases were subjected to dialysis (using a membrane with a cut-off of 12–14 kDa) and subsequently freeze-dried. Traces of phospholipids were eliminated by washing the samples with 90 % ethanol. The following references provide specific LPS purification methods for each samples: Francisella tularensis LVS [35]; LOS Moraxella catarrhalis [65,66]; Pseudomonas aeruginosa [67]; and S. meliloti (this study). It is worth noting that all the LPS samples used in this investigation originated from bacteria known to cause human diseases, apart from S. meliloti, a Gram-negative, non-pathogenic bacterium that is frequently present in soil. S. meliloti, commonly referred to as Rhizobium meliloti, has an LPS structure that is very different from other bacteria's due to the presence of lengthy fatty acyl chains [68]. Certain gut microbiota species, such Bacteroides and Prevotella, which possess physiologically inactive LPS serving as a *TLR4* ligand, also have extended fatty acyl chains, as a result, do not induce innate immune responses [69]. To serve as control samples, three distinct macromolecules were chosen due to their structural dissimilarity to LPS. These materials include chitin from crab shells (Sigma-Aldrich), lipoteichoic acid (LTA) from B. subtilis (Sigma-Aldrich), and peptidoglycan (PGN) from Gram-positive *S. aureus* (Sigma-Aldrich). LTA and PGN, derived from Gram-positive bacteria, exhibit distinct membrane compositions compared to Gram-negative bacteria, characterized by a thinner PGN layer beneath the LPS layer. In contrast, chitin is unique to fungi and insects and is not present in bacteria [70].

### 3.3 SERS spectra classification

A total of 5624 SERS spectra were acquired, produced by 11 bacterial endotoxins, chitin, lipoteichoic acid (LTA), and peptidoglycan (PGN). To improve the analysis's robustness, many preprocessing approaches were used. After traditional baseline correction approaches (e.g., polynomial fitting, Asymmetric Least Squares (ALS)), normalization, and data cleansing, additional preprocessing using adaptive deep learning-based baseline correction (e.g., Backprobagation) was used for comparison. This stage ensured an objective assessment of preprocessing approaches in order to validate *DeepRaman*'s pipeline.

**Fig. 8:**
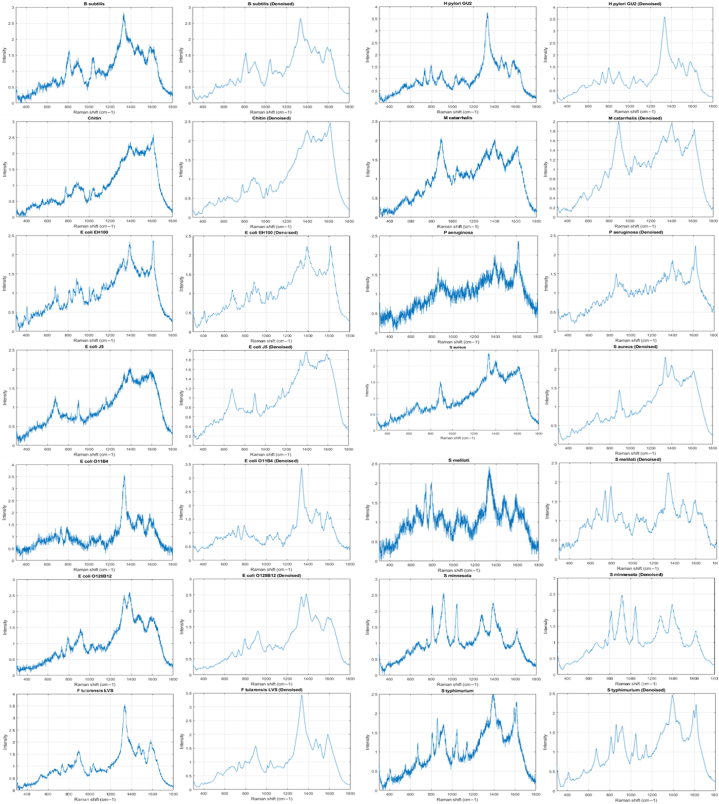
Pre-processed random SERS spectra for 14 bacteria.

**Fig. 9:**
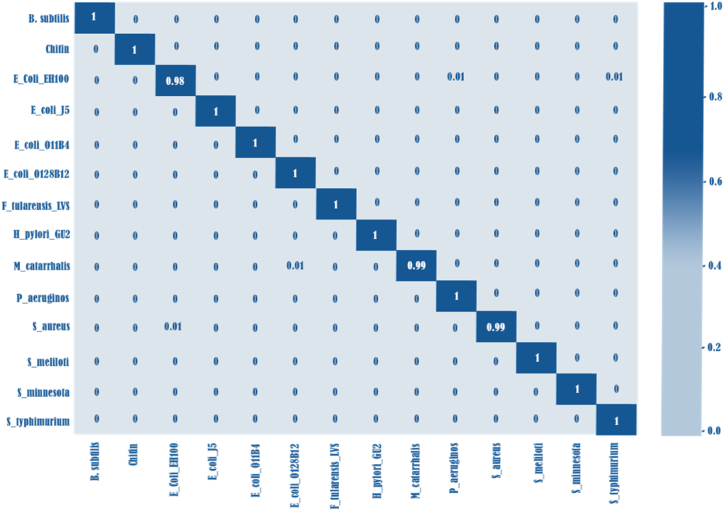
The confusion matrix of the DeepRaman model encompasses 11 LPS samples and 3 control samples. In this matrix, the entries denote the cases in which test spectra are classified by the DeepRaman model as pertaining to a particular class (in the first row), considering the actual class (in the first column). The entries on the diagonal represent the accuracies for each class.

We used our proposed filtering pre-processing as explained in step 2. Fig. 8 shows an example of pre-processed spectra for the 14 bacteria (see Fig. 9).

For this reason, the scikit-learn machine learning library in Python 3.8.3 was used. The testing set was kept aside to assess the predictive performance of the trained model, while the model training solely concentrating on the training spectral set. In the training stage, a method of five-fold cross-validation was used in order to maximize hyperparameters, which encompassed C (the parameter for regularization) and γ (the kernel value) for SVM, in addition to k (the neighbors quantity) for the algorithm of k-NN.

**Fig. 10:**
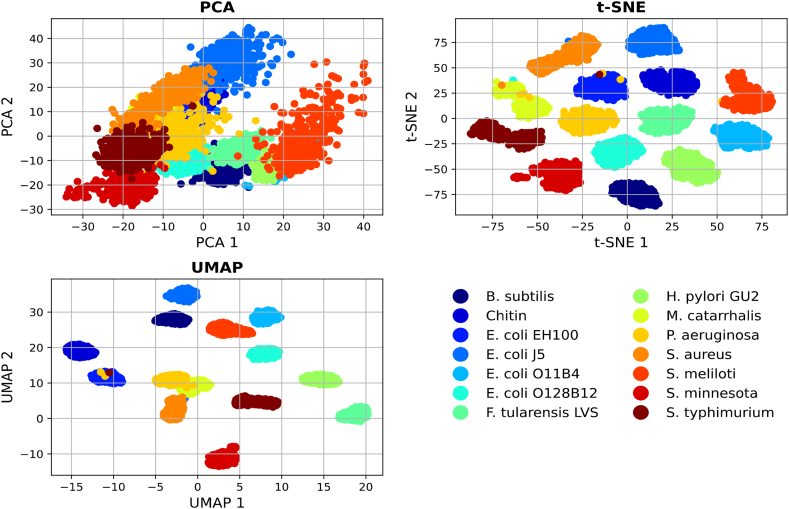
We can clearly see that all the chosen unsupervised machine learnings could distinguish between different samples in some extent. However, the t-SNE and UMAP have a relatively clearer separation between samples.

After cross-validation was finished, the best hyperparameters were selected, and accuracy was used to objectively evaluate the model's performance, sensitivity, and specificity metrics. Subsequently, the models underwent additional evaluation, during which their performance was tested on the external validation data from the testing set. By applying the models with optimal hyperparameters to the entire training set, feature importance was determined. the generation of the Receiver Operating Characteristic (ROC) curve and the production of the confusion matrix were conducted using the models to the testing spectral set. We evaluated the performance of k-NN on the testing spectral set by utilizing the trained k-NN classifiers. Fig. 10 illustrates the corresponding confusion matrix. Among the 3 reference samples and 11 LPSs, 11 were accurately classified (100 %), whereas a tiny percentage of spectra from *S. aureus*, M. catarrhalis, and E. coli-EH100 were incorrectly classified. Specifically, the precision for E. coli-EH100 attained 98.4 %, alongside 0.01 % of spectra misclassified as *P. aeruginosa* and 0.01 % as *S. typhimurium*. The accuracy for M. catarrhalis reached 99.0 %, with 0.01 % of spectra misclassified as *E. coli* O128:B12. Similarly, *S. aureus* achieved an accuracy of 99.0 %, with 0.01 % of spectra misclassified as E. coli-EH100.

We conducted a comparative analysis of *DeepRaman* against state-of-the-art methods for denoising and baseline correction. Han et al. (2024) demonstrated that AI-based methods, such as backpropagation (BP) and convolutional autoencoder (CDAE) [55], outperform traditional techniques like wavelet thresholding (WT) and adaptive iterative reweighted penalized least squares (airPLS). Using these methods alongside our algorithm, we evaluated their impact on classification accuracy with a consistent CNN classifier. *DeepRaman*, an end-to-end pipeline combining DenseNet classification with preprocessing via Fast Fourier Transform, showed superior performance in terms of SNR, MSE, and classification accuracy (Table 2).

**Table 2:** Results comparison of various baseline correction and denoising algorithms with their classification accuracy.

Preprocessing Method	SNR	MSE	Classification Accuracy (%) using CNN
Wavelet Transform (Sym7)	4.5 ± 1.2	0.045 ± 0.01	85.3 ± 2.2
Adaptive Iterative Reweighted. Penalized Least Squares (airPLS)	2.5 ± 0.8	0.075 ± 0.03	80.1 ± 4.5
CDAE	6.8 ± 1.5	0.025 ± 0.005	88.4 ± 3.1
Back propagation (BP)	N/A	N/A	82.3 ± 5.0
DeepRaman	12.1 ± 1.8	0.005 ± 0.002	99.0 ± 0.2

We can see that ML/AI based methods perform well in the term of SNR and MSE. CDAE obtained an SNR of 6.8 ± 1.5, surpassing traditional methods like Wavelet Transform (4.5 ± 1.2) and airPLS (2.5 ± 0.8) while airPLS and Wavelet transform exhibit higher MSE, reflecting less precise baseline corrections. Moreover, airPLS has the lowest SNR, suggesting it struggles to enhance the signal in noisy spectra. We can see also that *DeepRaman* achieves the lowest MSE at 0.005 ± 0.002, demonstrating its superior ability to accurately reconstruct the underlying signal. CDAE, a convolutional deep autoencoder also performs well, with an MSE of 0.025 ± 0.005, indicating relatively low distortion compared to the original signal.

The *DeepRaman* model is constructed upon an already-trained architecture that was trained using a large dataset comprising millions of images sourced from ImageNet. During the training process, a batch size of 256 was utilized for images with resolutions of 32x32 and 16x16, while a batch size of 64 was applied for images with a resolution of 64x64. This comprehensive training regimen spanned 235 epochs and encompassed a total of 17.9 million parameters. *DeepRaman* excels in enhancing the model's accuracy, achieving a remarkable 100 % multiclass accuracy, all while significantly expediting the training process, which involved 2 million FLOPs. Additionally, owing to its relatively shallow architectural design, *DeepRaman* boasts an impressively short inference time.

Furthermore, another distinctive characteristic contributing to *DeepRaman*'s superior performance lies in its complete independence from dimensionality reduction algorithms. In stark contrast to traditional methods, which invariably rely on feature compression techniques like PCA (Principal Component Analysis), *DeepRaman* inherently possesses the intrinsic capability to extract lower-dimensional representations directly from the data. To illustrate this inherent advantage, we present the compressed feature spaces obtained through PCA and *DeepRaman*. It is evident that all the selected unsupervised machine learning methods exhibited a certain degree of ability to differentiate between distinct samples. Nevertheless, t-SNE and UMAP, as indicated in Fig. 10, stand out by demonstrating a relatively more distinct separation between samples.

**Fig. 11:**
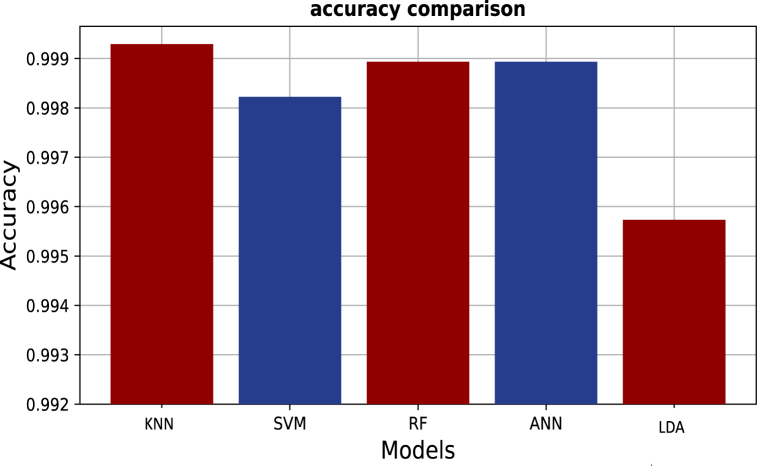
Performance of several ML on preprocessed SERS samples.

On the other hand, for the purpose of evaluating *DeepRaman*'s performance in contrast to other machine learning algorithms, we used RF, k-NN, SVM, ANN and LDA on the processed and reduced data. Fig. 11 shows the comparative accuracies of the other machine learning algorithm.

**Fig. 12:**
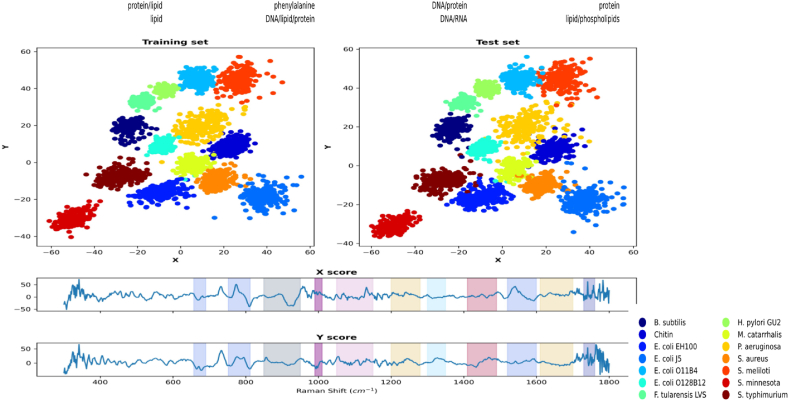
We observe that the DNA/RNA band in the range of 1500–1600 cm^−1^ plays a significant role in separating the samples along the x-axis, while phospholipids are more important for the distribution along the y-axis.

To project each spectrum into the latent layer, we calculated the dot product of the embedding space scores with the spectrum to find the associated score positions. This product's output can indicate which peak or section of the spectra is crucial in differentiating the spectra. Fig. 12 shows that the DNA/RNA band at 1500-1600 cm^−1^ separates samples along the x-axis, while phospholipids have a greater impact on distribution along the y-axis.

### 3.4 Inference *time*

Finally, training a Dense ResNet with a scalogram can take one to 2 h, based on the model's complexity, the dataset size, and the accessible computational resources. In our case, training a Dense ResNet on our dataset with six thousand of spectra and using a powerful GPU took around 0.5 h.

After using ½ hour for model training, the inference time for classifying new spectra is typically much shorter. It ranges from milliseconds to a few seconds per spectra.

## 4 Discussion

This study highlights the development and application of DeepRaman, a deep learning-based framework with an automated advanced spectral preprocessing and analysis for the classification of bacterial endotoxins, alongside its potential implications for medical diagnostics and other practical domains.

### 4.1 Technical improvements

We enhance the signal using an advanced technique that calculates the average between two signals, resulting in a signal with reduced high-frequency content compared to the original signals, which is particularly effective in managing difficult baseline scenarios that frequently cause failures. The processed signal is then converted into scalogram images, providing a detailed time-frequency decomposition representation, which are subsequently fed into a pre-trained CNN model (DenseCNN) to extract learned features for categorization purposes.

### 4.2 Practical applications

The findings demonstrate *DeepRaman*'s potential for rapid and accurate endotoxin detection, which is critical in medical diagnostics. For instance, this approach could be important for the early identification of sepsis, a life-threatening condition, enabling timely therapeutic interventions. Moreover, the capability to detect and classify endotoxins with such precision suggests applications in quality control, environmental monitoring, and pharmaceutical development, where trace detection of endotoxins is crucial.

## 5 Conclusion

In summary, our study conducted an in-depth SERS analysis on a total of fourteen bacterial endotoxins, using innovative AgNR substrates. This approach allowed for the identification of characteristic SERS peaks associated with these endotoxins, marking a successful achievement in our analysis.

To differentiate and categorize these diverse endotoxins, we employed a mixture of conventional machine learning algorithms and the novel deep learning approach known as *DeepRaman*. Through the implementation of suitable spectral preprocessing techniques and machine learning algorithms, most conventional methods achieved differentiation accuracy rates exceeding 98 %. Notably, *DeepRaman* consistently attained an accuracy of nearly 100 %, showcasing its superior performance in this context.

*DeepRaman*'s effectiveness stems from its unique architecture, inspired by the Progressive Fourier Transform and complemented by the scalogram transformation method. This innovative CNN operates autonomously, eliminating the need for human intervention and accommodating smaller datasets compared to traditional CNNs. Furthermore, *DeepRaman* excels in managing challenging baseline scenarios that often lead to failures with alternative methods, making it highly relevant for the promotion of widespread clinical adoption of Raman spectroscopy.

Our findings underscore the heightened sensitivity of *DeepRaman* in handling spectral shifts compared to conventional machine learning algorithms. This heightened sensitivity is expected to drive further advancements in Raman spectroscopy, particularly in the field of biomedical applications. Overall, our study demonstrates the capability of *DeepRaman* as an effective instrument for the swift and accurate detection of endotoxins, with significant implications for medical diagnoses and therapeutic decision-making, particularly in conditions such as sepsis.

Moreover, our study highlights the efficacy of using *DeepRaman* for the classification of several bacterial endotoxins. By leveraging this innovative approach, we achieved fast and remarkable accuracy rates in differentiating between endotoxin strains, surpassing traditional machine learning algorithms. Importantly, the use of *DeepRaman* significantly accelerates the classification process, offering a promising avenue for expediting the identification of bacterial endotoxins based on spectroscopy instead of using blood tests. This advancement holds significant potential for enhancing diagnostic speed and accuracy in the detection of endotoxin-related infections, ultimately improving patient outcomes.

## CRediT authorship contribution statement

**Samir Brahim Belhaouari:** Writing – review & editing, Methodology, Data curation. **Abdelhamid Talbi:** Formal analysis, Data curation. **Mahmoud Elgamal:** Investigation, Data curation. **Khadija Ahmed Elmagarmid:** Writing – review & editing, Visualization, Formal analysis. **Shaimaa Ghannoum:** Writing – review & editing, Data curation. **Yanjun Yang:** Resources, Investigation, Data curation, Conceptualization. **Yiping Zhao:** Methodology, Funding acquisition, Conceptualization. **Susu M. Zughaier:** Writing – review & editing, Project administration, Investigation, Funding acquisition, Conceptualization. **Halima Bensmail:** Writing – review & editing, Writing – original draft, Supervision, Project administration, Methodology, Conceptualization.

## Data availability

Data are available on reasonable request.

## Author disclosures

The author reports no conflicts of interest in this work.

## Declaration of Generative AI and AI-assisted technologies in the writing process:

I declare that I did not use GenAI and AI to write this manuscript.

## Funding

This research was funded by the Qatar National Research Fund (10.13039/100008982QNRF now QRDI) under Grant NPRP12S-0224-190144. Computational resources were provided by the Qatar Research Computing Institute (QCRI).

## Declaration of competing interest

The authors declare that they have no known competing financial interests or personal relationships that could have appeared to influence the work reported in this paper.
