# Supplementary material for: DeepRaman: Implementing surface-enhanced Raman scattering together with cutting-edge machine learning for the differentiation and classification of bacterial endotoxins
Source: Heliyon. 2025 Feb 8;11(4):e42550. doi: 10.1016/j.heliyon.2025.e42550 (PMC11870271; doi:10.1016/j.heliyon.2025.e42550)
Supplement: Multimedia component 1 [file mmc1.docx]

**Title:** DeepRaman: Implementing Surface-Enhanced Raman Scattering Together with Cutting-Edge Machine Learning for the Differentiation and Classification of Bacterial Endotoxins.

**Belhaouari, S. et al.**

**Supplementary** **Materials:**

**Details on Data Preprocessing (step 2):**

From the signal amplitude variation of the signal $x\left( n \right)$, upper and lower bound signal will be extracted to form an envelope of the signal, $x_{U}(n)$ and $x_{L}(n)$ respectively. The first average signal between upper and lower signal,$x_{1}\left( n \right)$, will have fewer high frequencies compared to the original signal. This process can be reapplied again on $x_{1}\left( n \right)$to get the signal $x_{2}\left( n \right)$ and so on iteratively to remove high frequencies further.


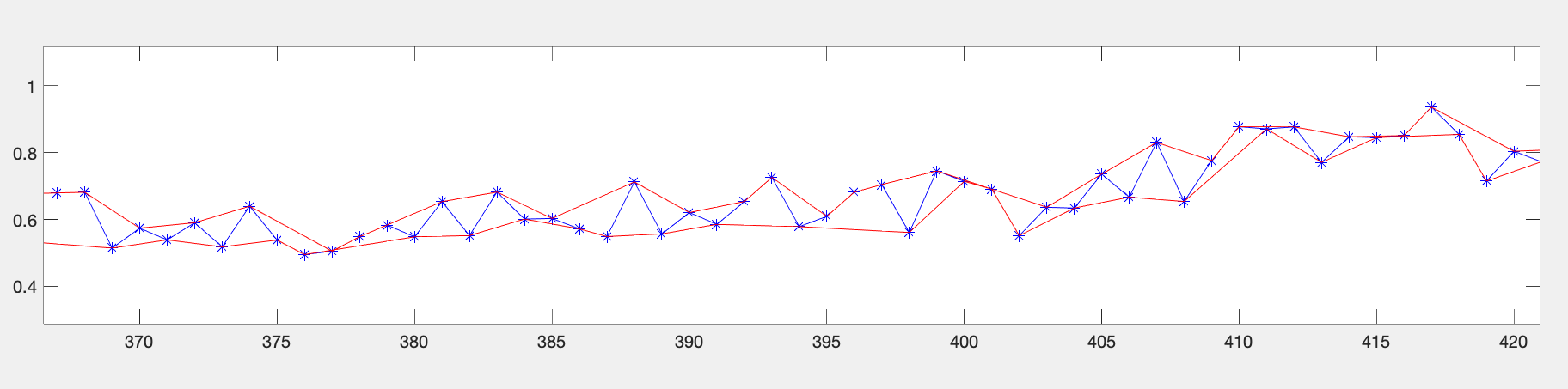


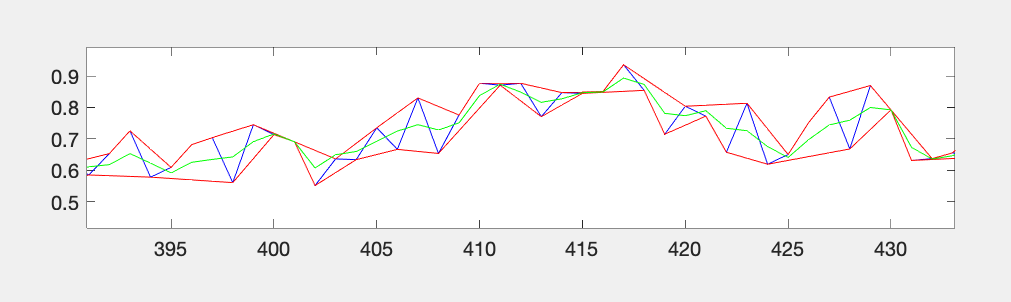

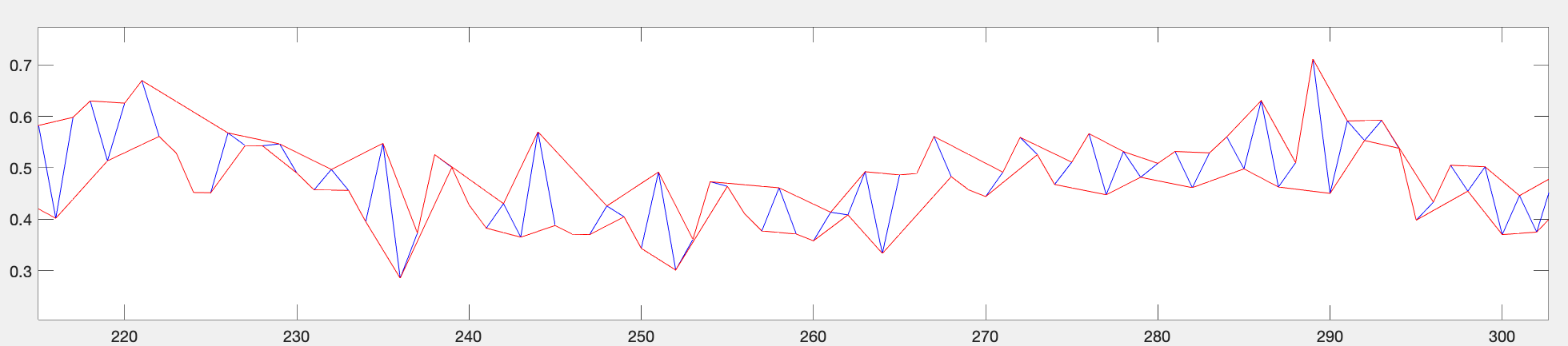


Figure S1: Upper and lower bound signal are extracted to form an envelope of the signal respectively. The first average signal between upper and lower signal and average is calculated.

The upper signal is egal to the original signal at the point *i* when the derivative is positive i.e., $x\left( i+1 \right)-x(i)\geq0$, and the lower signal is egal to the original signal at the point *i* when the derivative is positive i.e., $x\left( i+1 \right)-x\left( i \right)<0$, and the lower. The missing points will be calculated by simple linear interpolation between two closest know points.

The upper signal can be defined mathematically as follows:

$x_{U}\left( i \right)=\left\{ \begin{aligned} x\left( 0 \right) if i=0 \\ x\left( i \right) if x\left( i+1 \right)\geq x\left( i \right) \\ \frac{\left( x\left( j_{i+1} \right)-x\left( j_{i} \right) \right)}{j_{i+1}-j_{i}}\left( j_{i}-i \right)+x\left( j_{i} \right) if x\left( i+1 \right)<x\left( i \right) \\ x\left( N \right) if i=N \end{aligned} \right.$ (1)

where

$j_{i}=0\wedge\min_{j} \{j:x\left( j \right)\geq x\left( j+1 \right)\& j<i\}$ and $j_{i+1}=N\vee\min_{j} \{j:x\left( j \right)\geq x\left( j+1 \right)\& j>i\}$

Same idea, we can formulate the lower signal as follows:

$x_{L}\left( i \right)=\left\{ \begin{aligned} x\left( 0 \right) if i=0 \\ x\left( i \right) if x\left( i+1 \right)<x\left( i \right) \\ \frac{\left( x\left( j_{i+1} \right)-x\left( j_{i} \right) \right)}{j_{i+1}-j_{i}}\left( j_{i}-i \right)+x\left( j_{i} \right) if x\left( i+1 \right)\geq x\left( i \right) \\ x\left( N \right) if i=N \end{aligned} \right.$ (2)

where

$j_{i}=0\wedge\min_{j} \{j:x\left( j \right)<x\left( j+1 \right)\& j<i\}$ and $j_{i+1}=N\vee\min_{j} \{j:x\left( j \right)<x\left( j+1 \right)\& j>i\}$


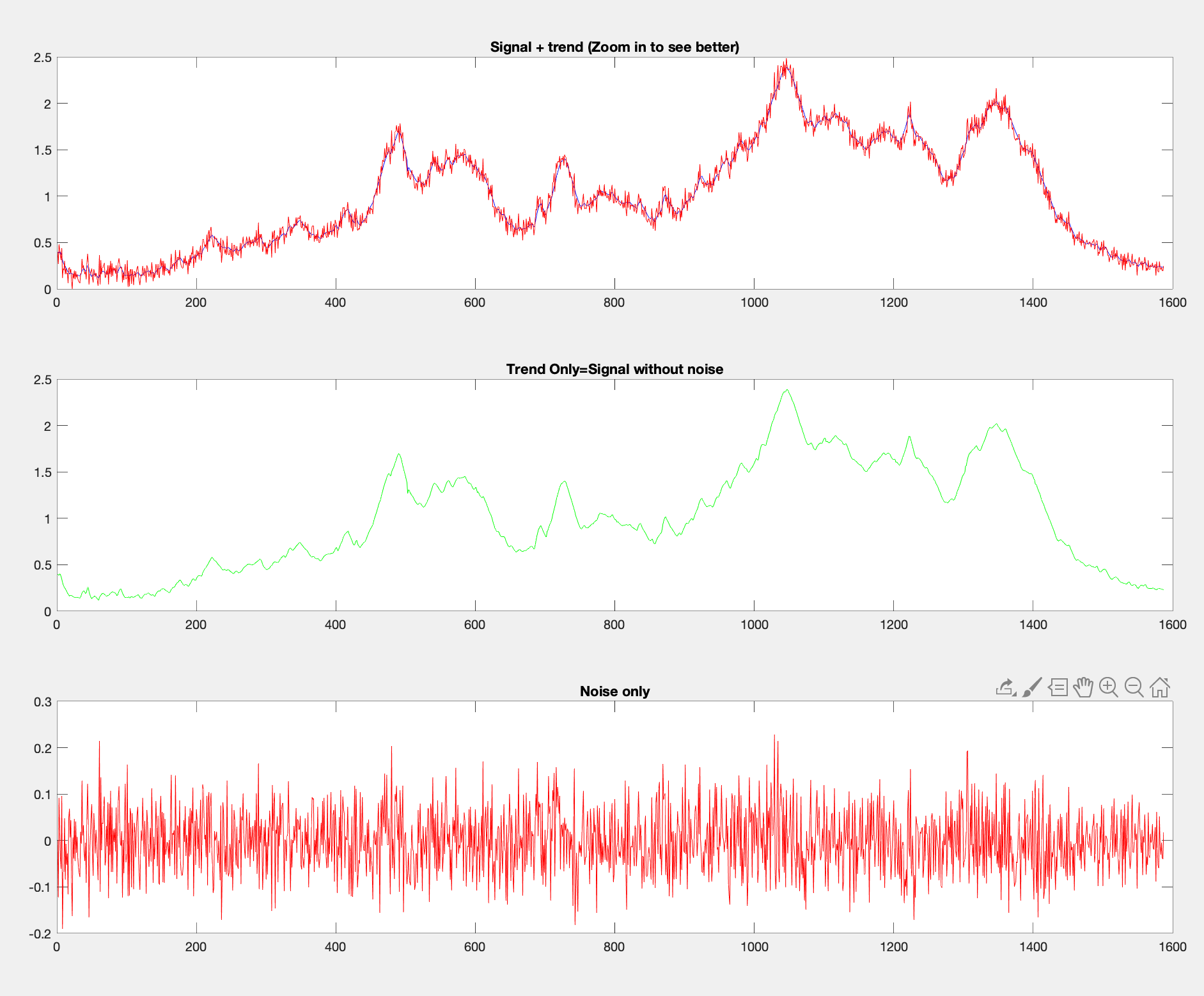


Figure S2: Extracting Trend and noise from a signal+trend spectra.

**Details on transformation of the spectra using Progressive Fourier Transform (step 3):**

Fourier Transform comes in different form depending on the domain of application. We mention Continuous Fourier Transform (CFT) applied to continuous signal, Discrete Fourier Transform (DFT) which is calculated at discrete frequencies. Because the calculation of the DFT for large signals with large size $N$ is time-consuming and consumes $O(N^{2})$. Thus, Fast Fourier Transform (FFT) only considers sample point intervals N as a power of two $N = 2^{m}, m \in N$ and consumes $O(N log N)$.


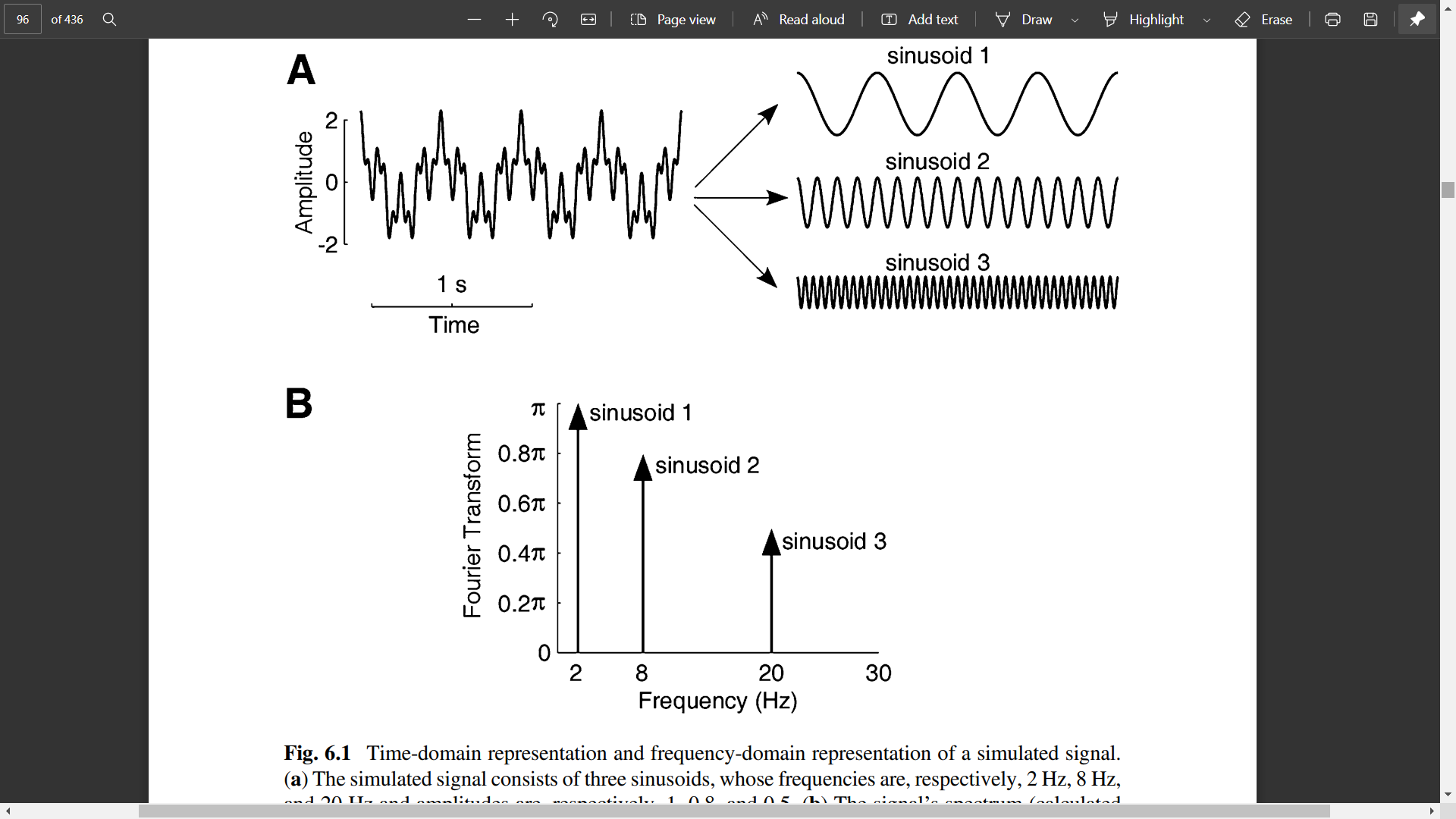


Figure S3: The representations of the signal in (a) time-domain and (b) frequency-domain [87]
